# Supplementary material for: Integrative genotyping of cancer and immune phenotypes by long-read sequencing
Source: Nat Commun. 2024 Jan 2;15:32. doi: 10.1038/s41467-023-44137-7 (PMC10762175; doi:10.1038/s41467-023-44137-7)
Supplement: Supplementary file 1 — Supplementary Information [file 41467_2023_44137_MOESM1_ESM.pdf]

Supplementary Data

Integrative genotyping of cancer and immune phenotypes by long-read sequencing.

Inventory:

- Supplementary Figures 1-14
- Supplementary Tables 1-10
- Supplementary References

Supplementary Figures

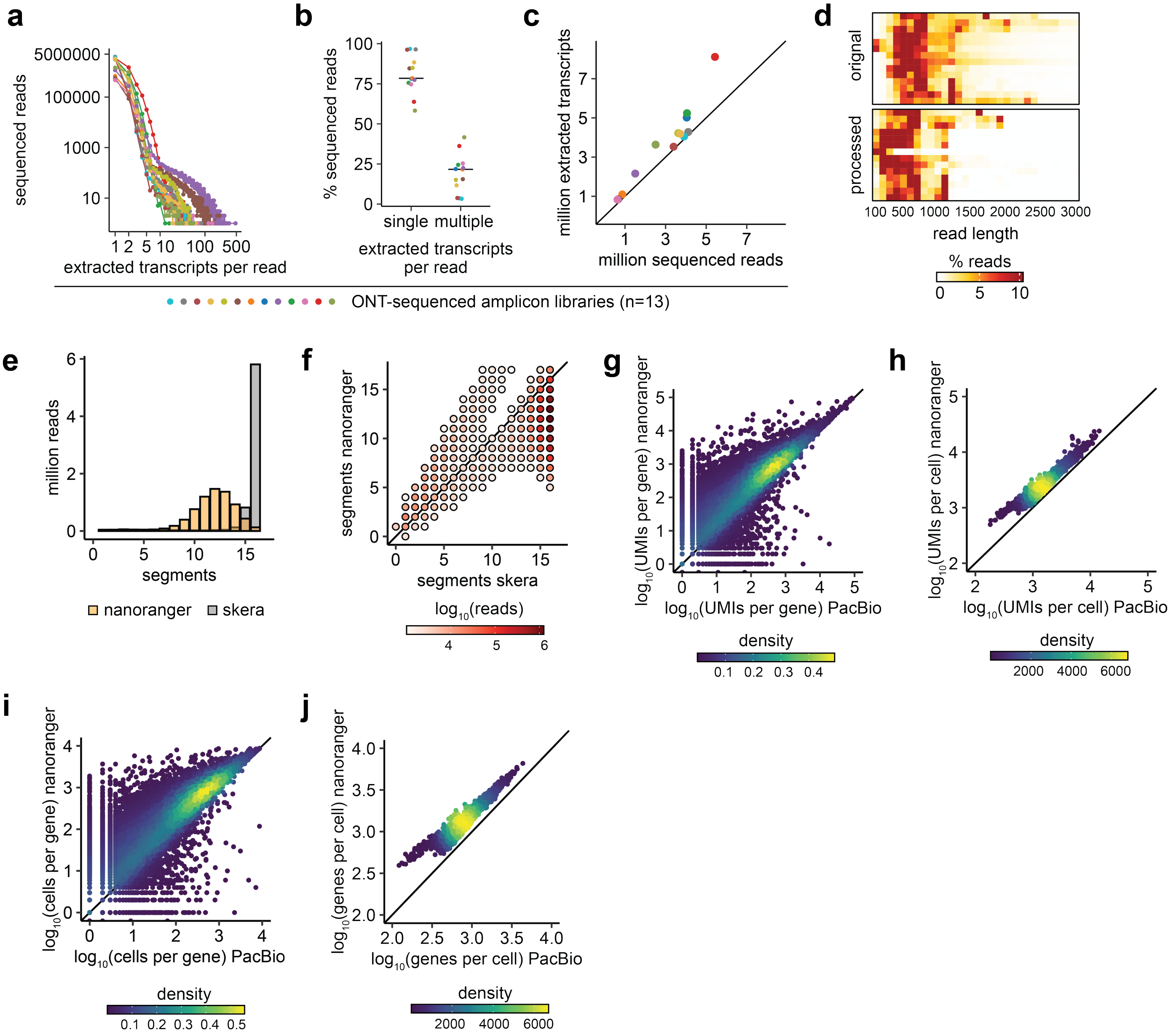

Supplementary Figure 1. Benchmarking of *nanoranger*.
a Distribution of number of extracted transcripts (defined by a truseq motif and template-switch oligo (TSO) sequence at a distance of 26 nucleotides) per sequenced read for 13 long-read sequenced amplicon libraries.
b Percentage of sequenced reads containing a single or multiple extracted transcripts.
c Number of extracted transcripts compared to number of sequenced reads.
d Length of reads (original) and extracted transcripts (processed) for analysis of somatic mutations.
e, f Synthetically concatenated data sequenced on PacBio were processed with *nanoranger* and compared to results of the PacBio processing pipeline. Number of segments extracted from each read with *nanoranger* and skera.
g, h Number of identified molecules per gene (g) and per cell (h) with *nanoranger* and the PacBio analysis pipeline.
i, j Number of cell barcodes associated with genes and number of genes detected per cell barcode with *nanoranger* and the PacBio analysis pipeline.

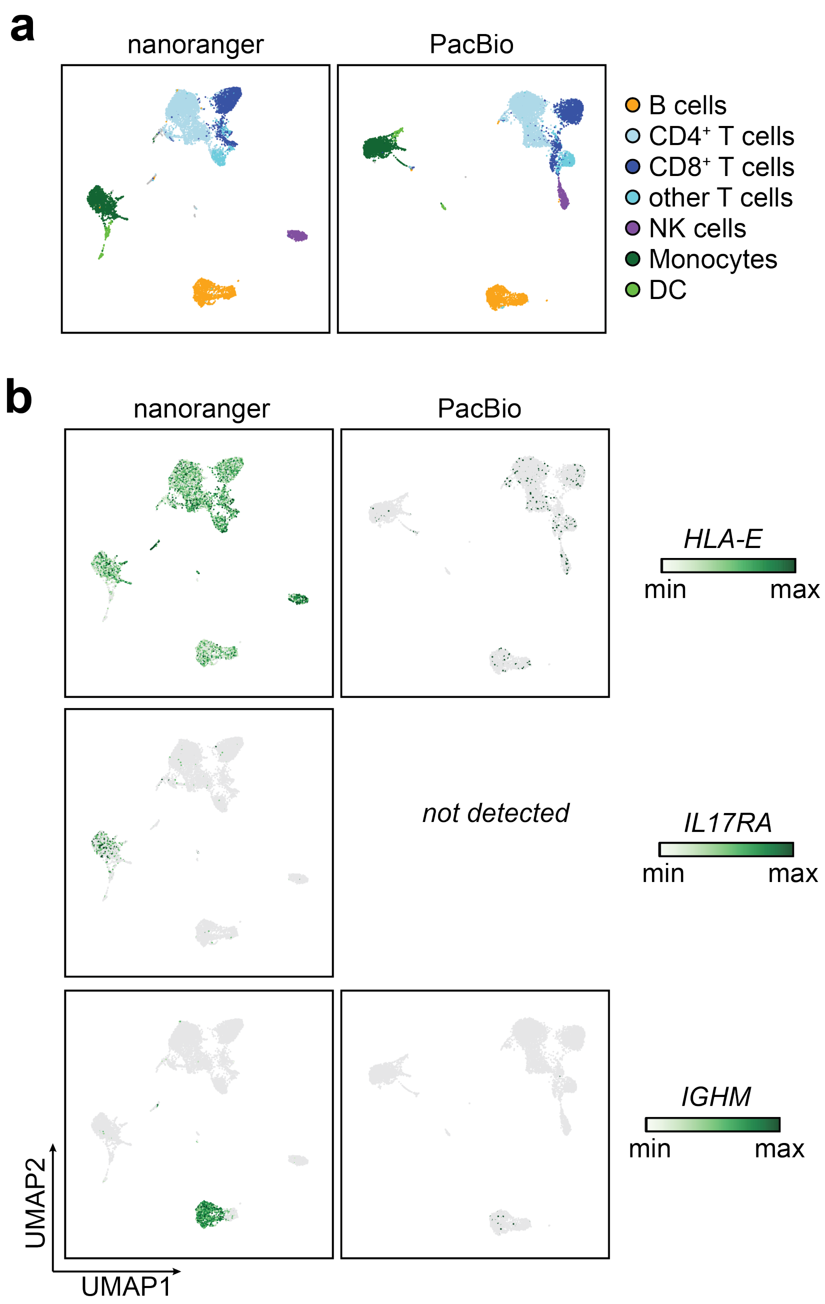

Supplementary Figure 2. Analysis of cell types and gene expression profiles using deconcatenated PacBio data.

a, b UMAP projection of cell types identified based on count matrices generated with *nanoranger* or the PacBio analysis pipeline using PacBio data (a). The feature plots demonstrate genes with higher detection rate using *nanoranger* processing (b).

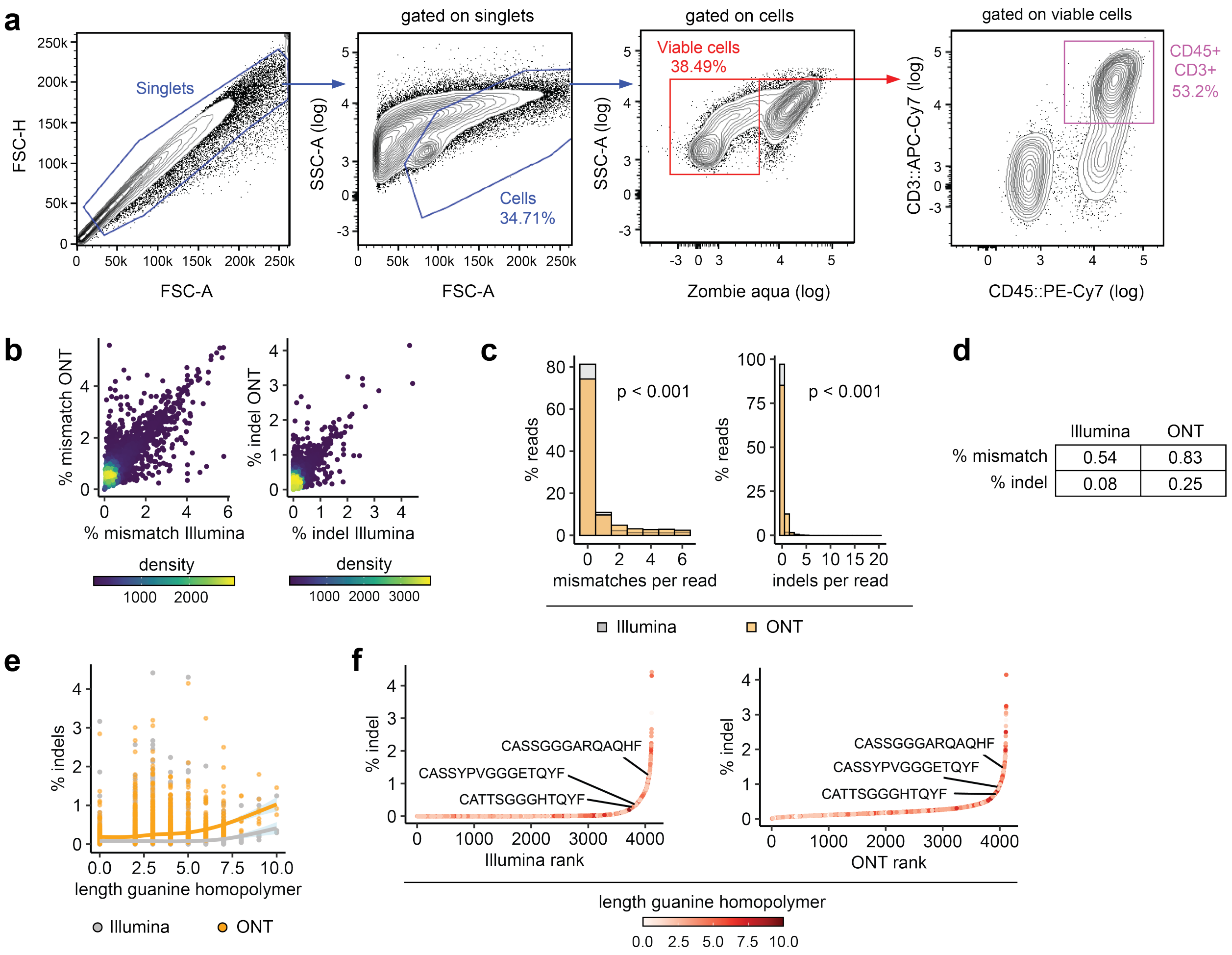

Supplementary Figure 3. Comparison of sequencing error with Illumina and V14 Oxford Nanopore Technology (ONT) chemistry based on T cell receptor (TCR) sequencing data.

a Gating strategy of tumor-infiltrating lymphocytes (TILs) used for single cell TCR sequencing. The TCR data of TILs were obtained by resequencing an amplicon library using the Oxford Nanopore platform that had previously been reported (Oliveira et al., Nature 2021).[1]

b TCR reads were aligned against their respective consensus sequence, enabling to determine mismatch and indel rates of each read.

c Per-base mismatch rate (left) and indel rate (right) of each read with Illumina and ONT sequencing.

d Distribution of mismatches and indels per read for Illumina (grey) and ONT (orange).

e Statistics of mismatch and indel rates with Illumina and ONT.

f Rate of indels with Illumina (grey) and ONT (orange) increases with higher lengths of guanine homopolymers in TCR reads.

Statistical testing with two-sided Student's t-test.

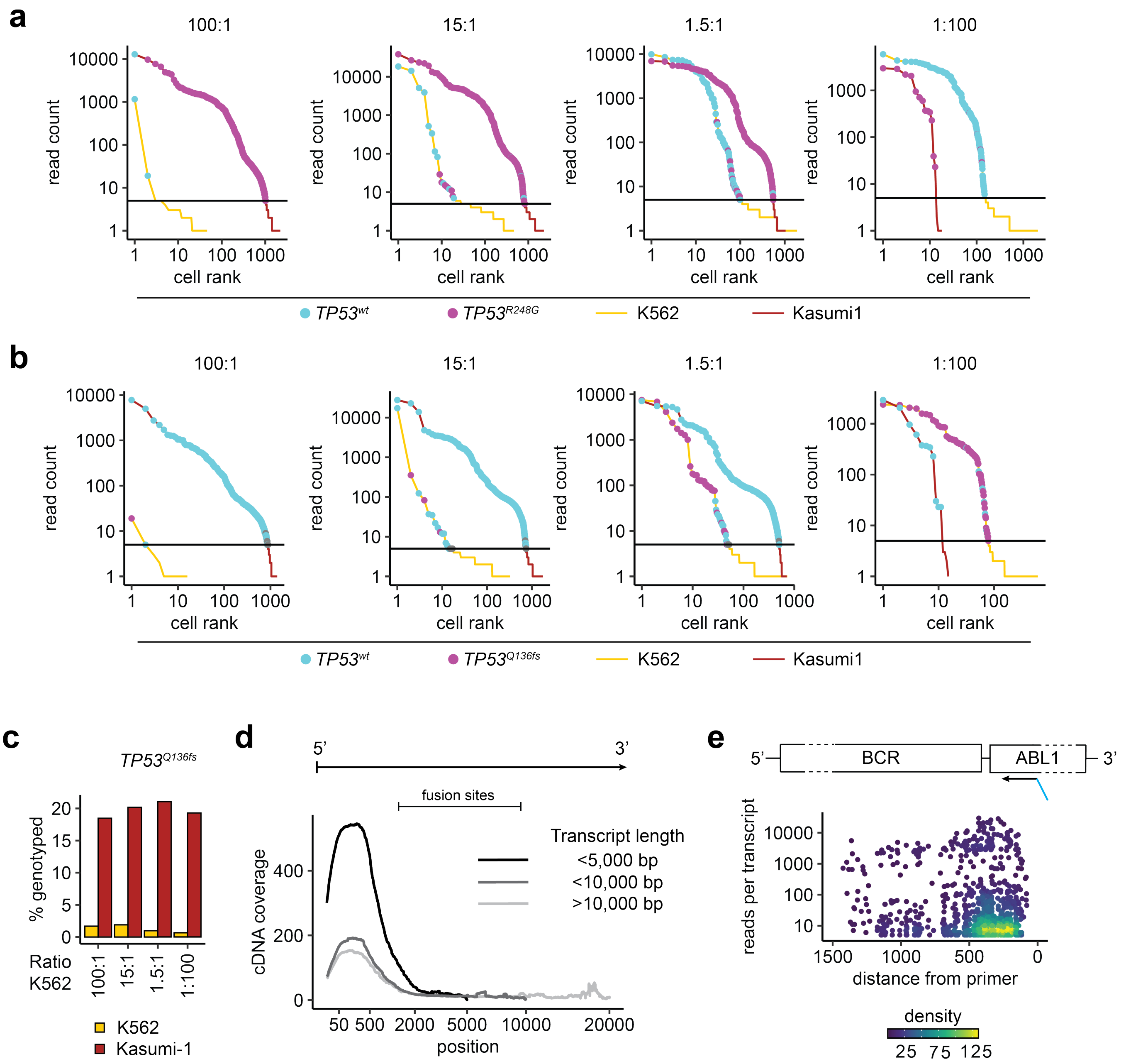

Supplementary Figure 4. Detection of somatic mutations and fusions in Kasumi-1 and K562.
a, b Knee plots for mixing experiment of Kasumi-1 and K562 cells. Read count for each cell barcode is shown for all cells ranked by their read count for Kasumi-1 (red) and K562 (yellow). Detection of *TP53*^{R248G} (top) or *TP53*^{Q136fs} (bottom) is shown in purple, while wildtype cells are shown in cyan.
c Percentage of genotyped cells for *TP53*^{Q136fs} for K562 (yellow) and Kasumi-1 (red).
d Coverage of 5' 10x cDNA after removal of template-switch oligo (TSO) artifacts.
e Amplification rate versus read length for reads mapping to *BCR::ABL1* in K562.

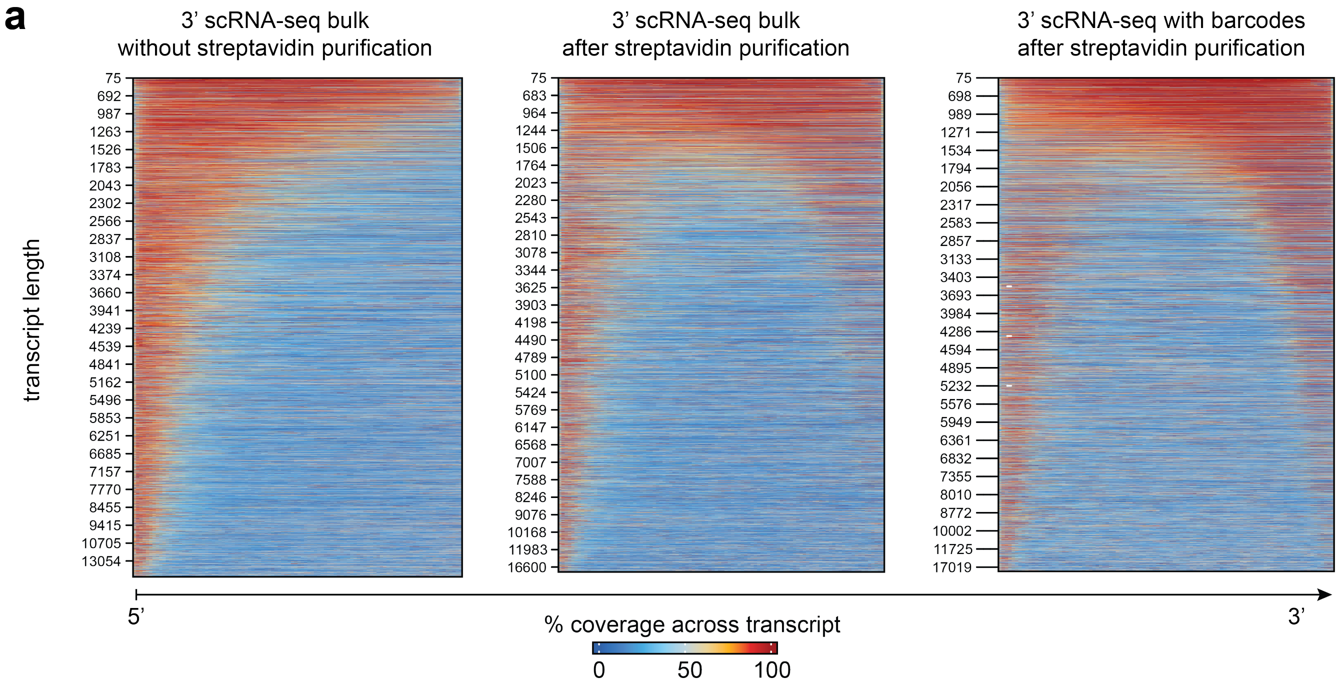

Supplementary Figure 5. Coverage of 3’ 10x Genomics single cell cDNA libraries.
a Coverage of 10x 3’ cDNA Oxford Nanopore sequencing reads in pseudobulk analysis following processing with *nanoranger* without streptavidin purification (left), after streptavidin purification (middle) and after streptavidin purification and extraction of reads containing a cell barcode (right).

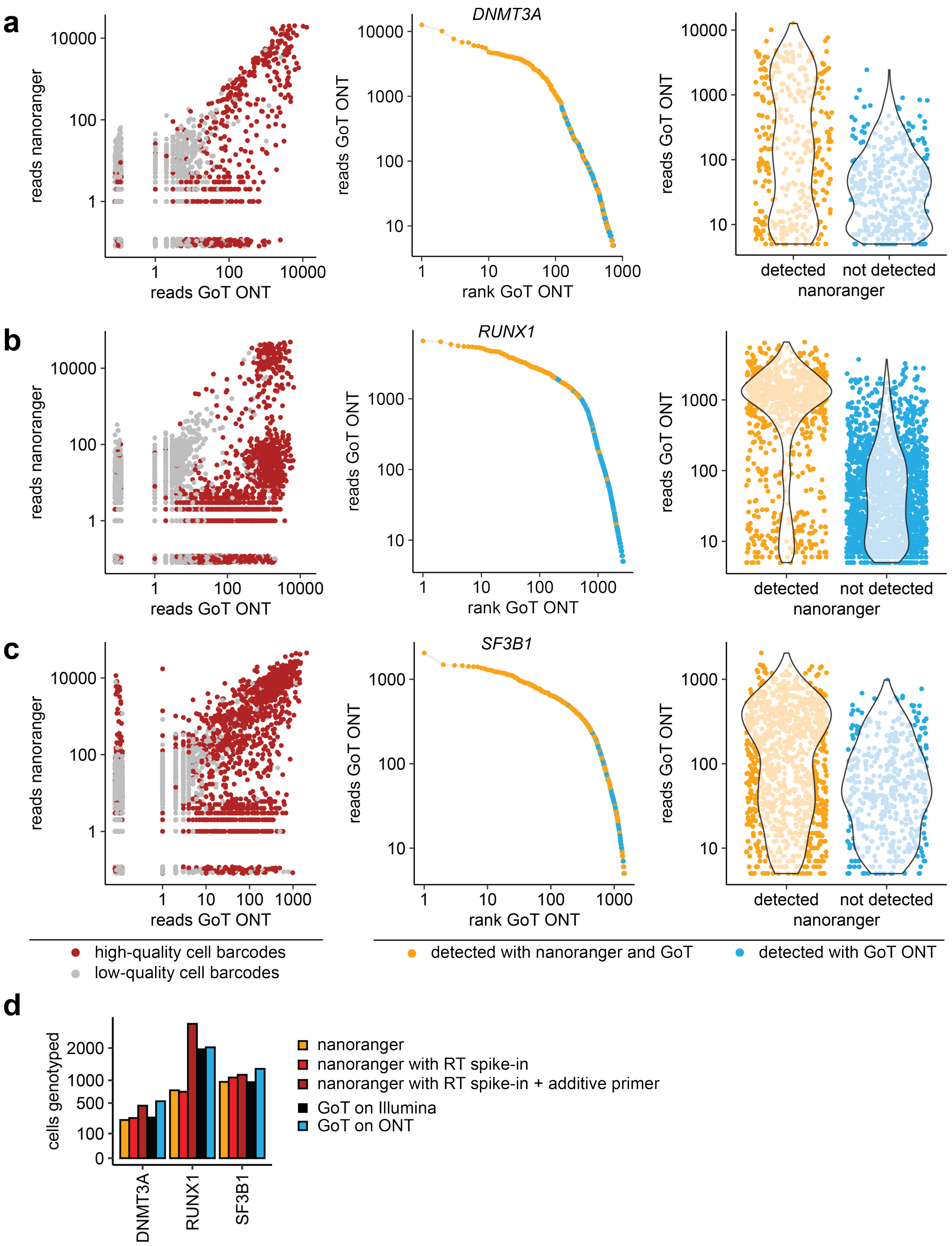

Supplementary Figure 6. Comparison of sequencing coverage with *nanoranger* and GoT ONT reveals that GoT improves capture of lowly abundant transcripts.

a-c Comparison of sequencing depth per cell barcode (high-quality cell barcode – red, low-quality cell barcode – grey) between *nanoranger* and genotyping of transcriptomes (GoT) sequencing using Oxford Nanopore (ONT) (left). Knee plots and violin plots showing sequencing depth of high-quality cell barcodes detectable with nanoranger and GoT (yellow) or only with GoT ONT (blue) (middle and right). To enable direct comparison of captured cell barcodes, the cDNA for the GoT condition was used as input for nanoranger.

d Number of cells genotyped with each experimental condition. RT – reverse transcription.

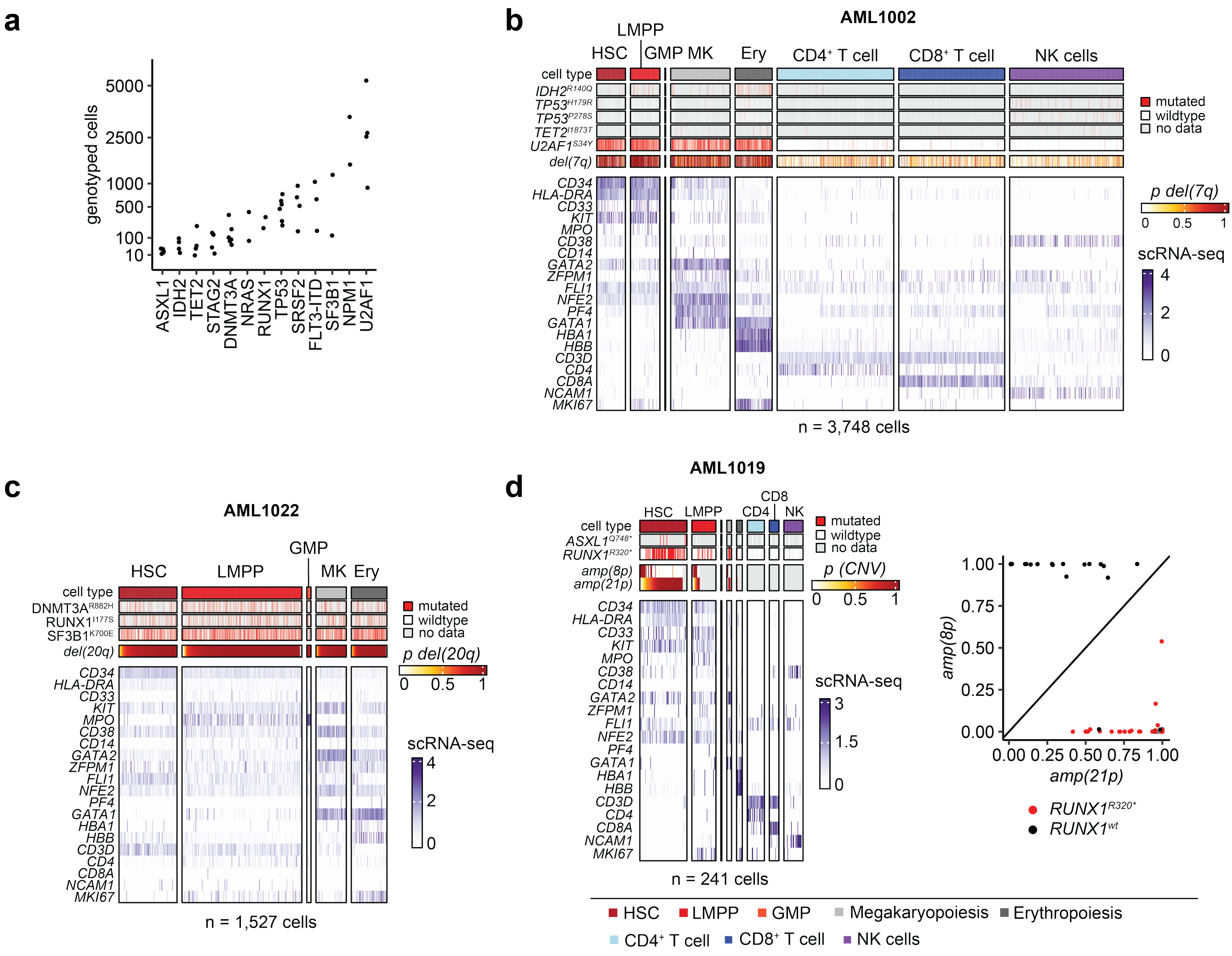

Supplementary Figure 7. Detection of leukemic clones in erythroid and megakaryocytic cells.

a Genotyping rate of different recurrently mutated genes in AML/MDS. Shown are the number of cell barcodes that are associated with high-quality cells.

b Integrated analysis of somatic mutations (*IDH2*^{R140Q}, *TP53*^{R179H}, *TP53*^{P278S}, *TET2*^{I1873T}, *U2AF1*^{S34Y}), detection of *del(7q)* and gene expression across hematopoietic cell types in AML1002.

c Detection of somatic mutations (*DNMT3A*^{R882H}, *RUNX1*^{I117S}, *SF3B1*^{K700E}), *del(20q)* and RNA expression across hematopoietic cell types in AML1022.

d Detection of somatic mutations (*ASXL1*^{Q748*}, *RUNX1*^{R320*}), chromosomal aberrations (*amp(8)* and *amp(21p)*) and RNA expression across hematopoietic cell types in AML1019. The scatter plot (right) demonstrates mutual exclusivity of two AML subclones defined by *amp(8p)* and *amp(21p)*, consistent with detection of *RUNX1*^{R320*} and wildtype *RUNX1*^{wt}.

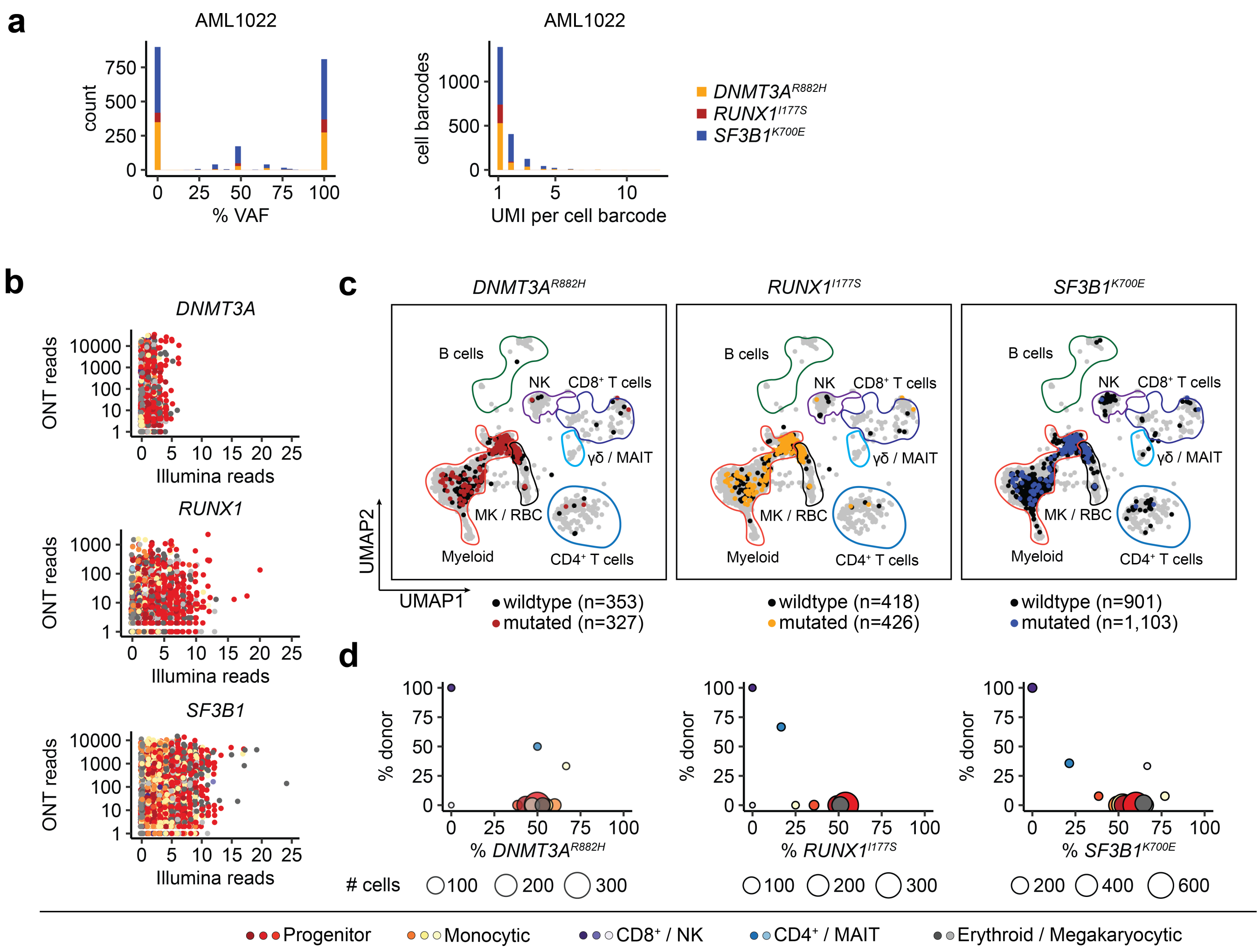

Supplementary Figure 8. Limits of targeted genotyping.

a Distribution of cell numbers across apparent variant allele frequencies per cell for *DNMT3A*^{R882H}, *RUNX1*^{I177S} and *SF3B1*^{K700E} mutation (left) and distribution of UMIs detected per cell barcode (right) in AML1022.

b Read coverage of *DNMT3A*, *RUNX1* and *SF3B1* transcripts achieved with *nanoranger* versus whole-transcriptome Illumina sequencing.

c Mapping of *DNMT3A*^{R882H}, *RUNX1*^{I177S} and *SF3B1*^{K700E} mutations to hematopoietic cell types in bone marrow of AML1022.

d Comparison of pseudobulk donor chimerism and variant allele frequency for *DNMT3A*^{R882H}, *RUNX1*^{I177S} and *SF3B1*^{K700E} mutations across detected cell types in AML1022 bone marrow. The size of bubbles indicates the number of cells per cell type.

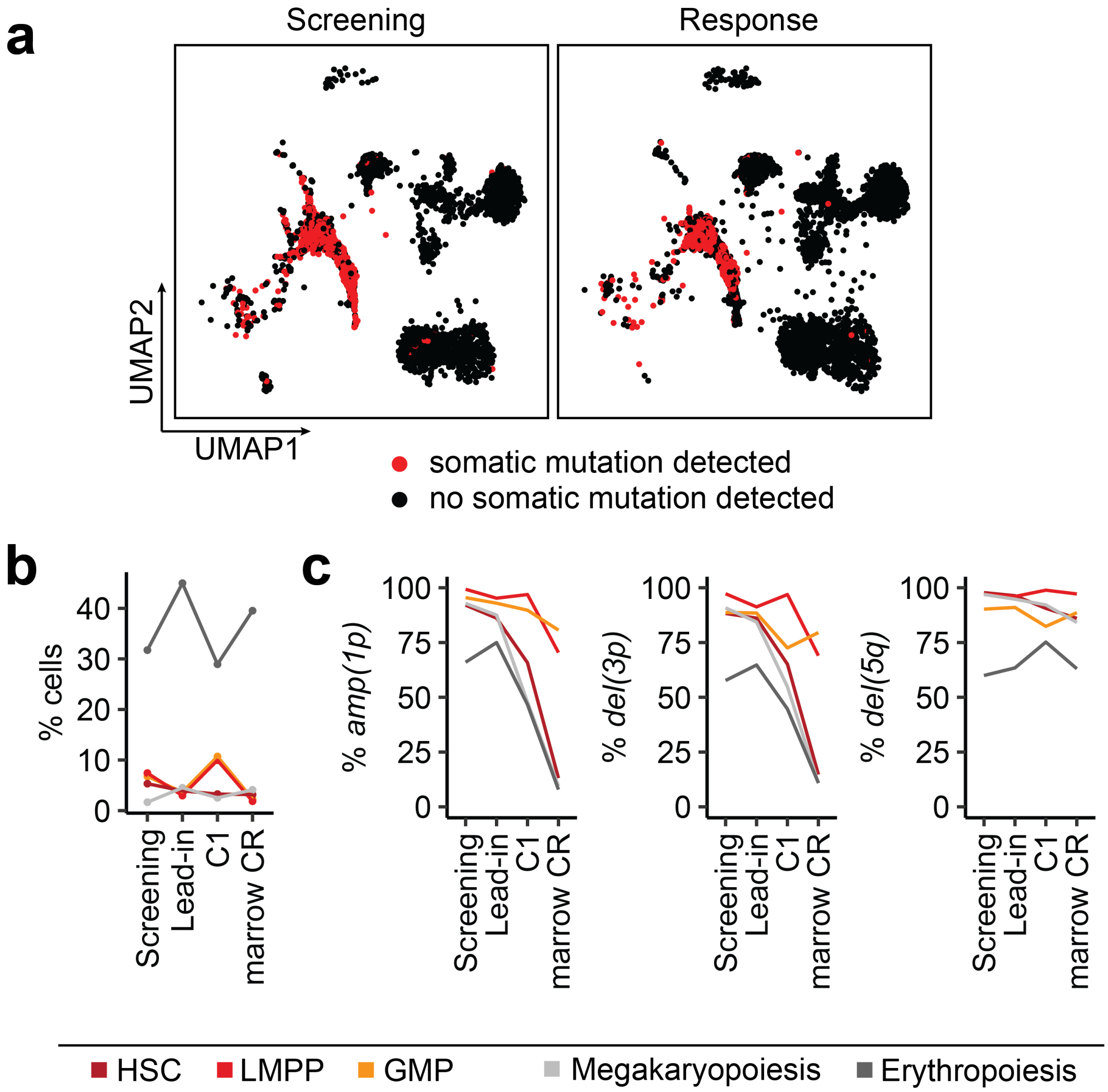

Supplementary Figure 9. Longitudinal tracking of somatic mutations in AML.
a Detection of recurrent somatic mutations at screening (left) and at time of response (right) in 5 study participants of ETCTN/CTEP 10026. The UMAP projection shows cells with wildtype (black) and mutated alleles (red).
b, c Percentage of cells (b) and percentage of cells with *amp(1p), del(3p)* or *del(5q)* throughout treatment (c) across HSC, LMPP, GMP, megakaryopoietic and erythroid compartment in AML8007.

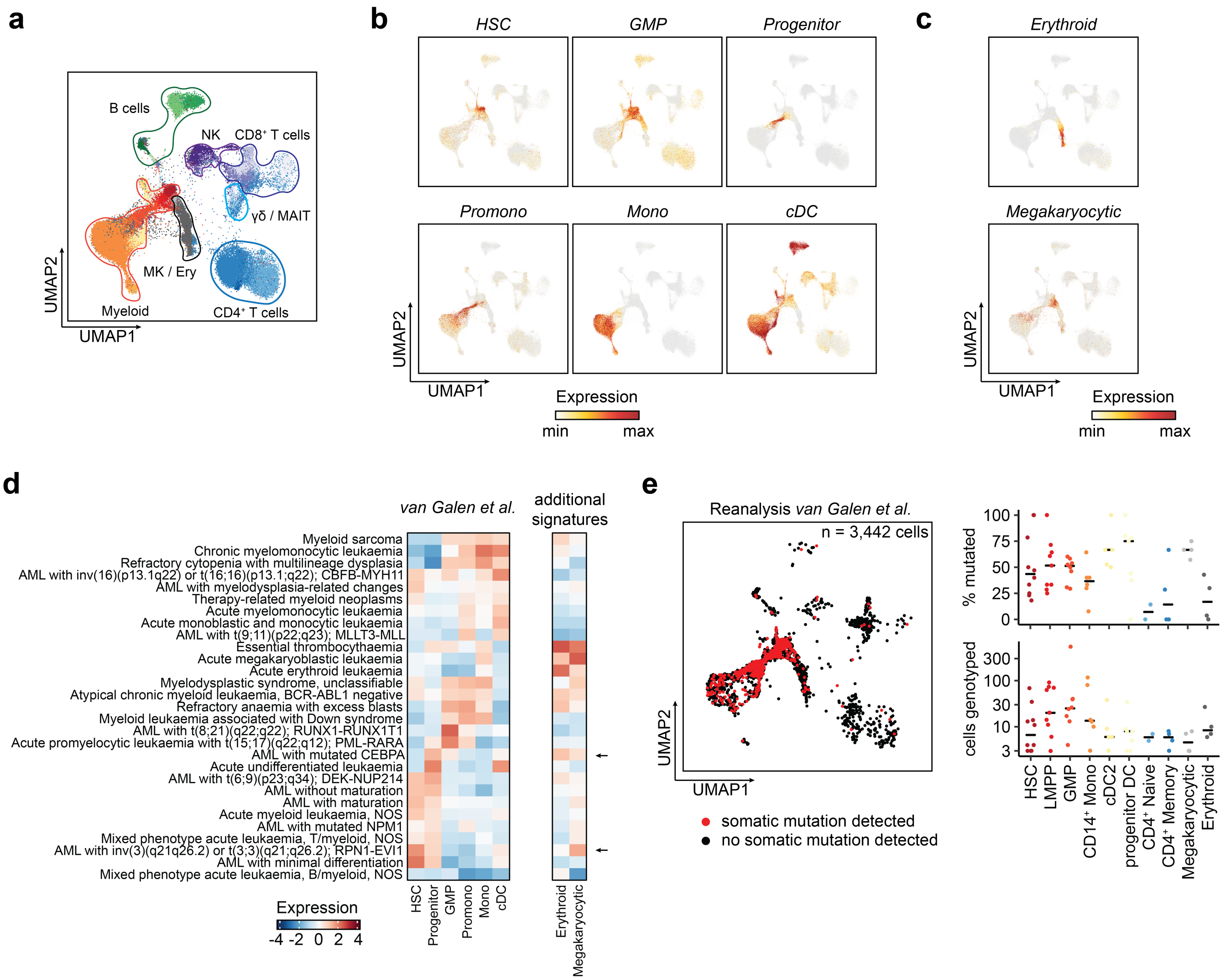

Supplementary Figure 10. Expression clusters in AML.

a-d Overview of UMAP projection of bone marrow-derived AML single cell RNA sequencing (scRNA-seq) profiles. Application of van Galen[2] (b) and erythroid/megakaryocytic signature (c) to AML bone marrow scRNA-seq profiles and to 646 bulk RNA-seq profiles obtained from the Beat AML dataset (d).[3]

e Re-analysis of data from *van Galen* et al. Cell types were identified using reference-based annotation and projected onto reference UMAP (Methods). Percentage of cells with detectable somatic mutation (top) and number of cells that could be genotyped per cell type (bottom).

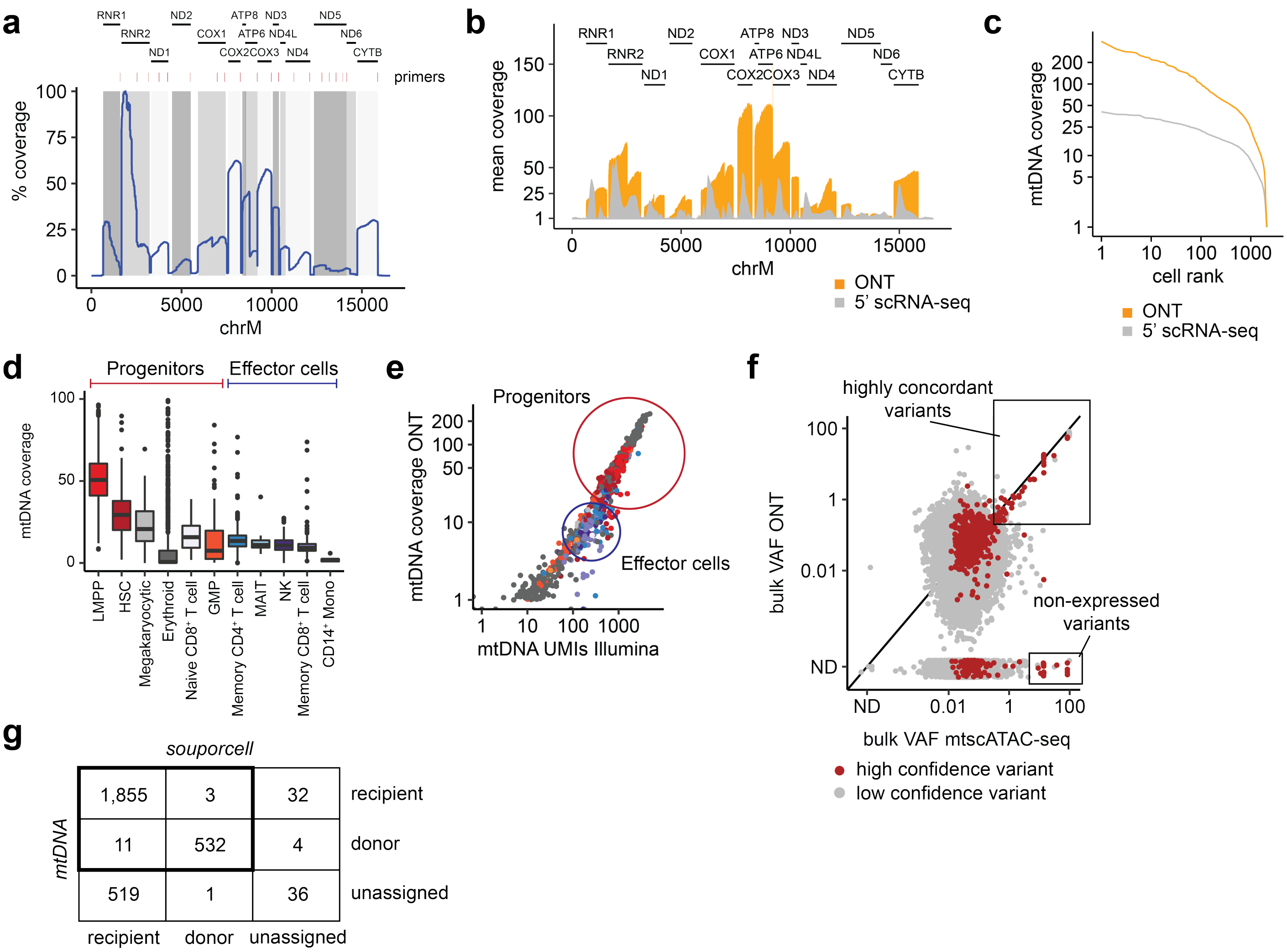

g

souporcell (columns) vs. *mtDNA* (rows)

mtDNA \ *souporcell*	recipient	donor	unassigned
recipient	1,855	3	32
donor	11	532	4
unassigned	519	1	36

Supplementary Figure 11. Tracking of somatic and mitochondrial DNA mutations.
a Native coverage of mitochondrial transcripts in 5' scRNA-seq cDNA after removal of template-switch oligo artifacts as sequenced by long-read sequencing. Mitochondrial genes are annotated. Primers used for amplification of mitochondrial genes are indicated by red vertical lines.
b Targeted amplification of mitochondrial transcripts increases their coverage compared to standard whole-transcriptome 5' scRNA-seq libraries.
c Comparison of mitochondrial transcript coverage with targeted amplification using Oxford Nanopore sequencing (ONT) (yellow) and from native whole-transcriptome 5' scRNA-seq library (grey) from the same sample.
d Mitochondrial transcripts are more abundant in metabolically active cells such as most AML populations (progenitors) compared to T/NK cells (effector cells) and therefore are preferentially amplified.
e Coverage of mitochondrial transcripts after targeted amplification and Oxford Nanopore sequencing with *nanoranger* (ONT) correlates with coverage from standard whole-transcriptome 5' scRNA-seq data (Illumina). Progenitor cells with higher metabolic activity have higher mitochondrial DNA coverage than effector cells.
f Mitochondrial DNA mutations with bulk variant allele frequency >0.5% show high agreement between scRNA-seq and mtscATAC-seq libraries. As mtscATAC-seq is DNA-based, it also detected non-expressed mtDNA mutations that are part of the hypervariable region but undetectable using scRNA-seq, as indicated in the lower box.
g Concordance of donor and recipient annotation with mtDNA variants and *souporcell*[4]. The relevant fields are highlighted. Due to insufficient coverage of cells with little mtDNA abundance, mtDNA variants are unable to assign some cells that *souporcell* can annotate.

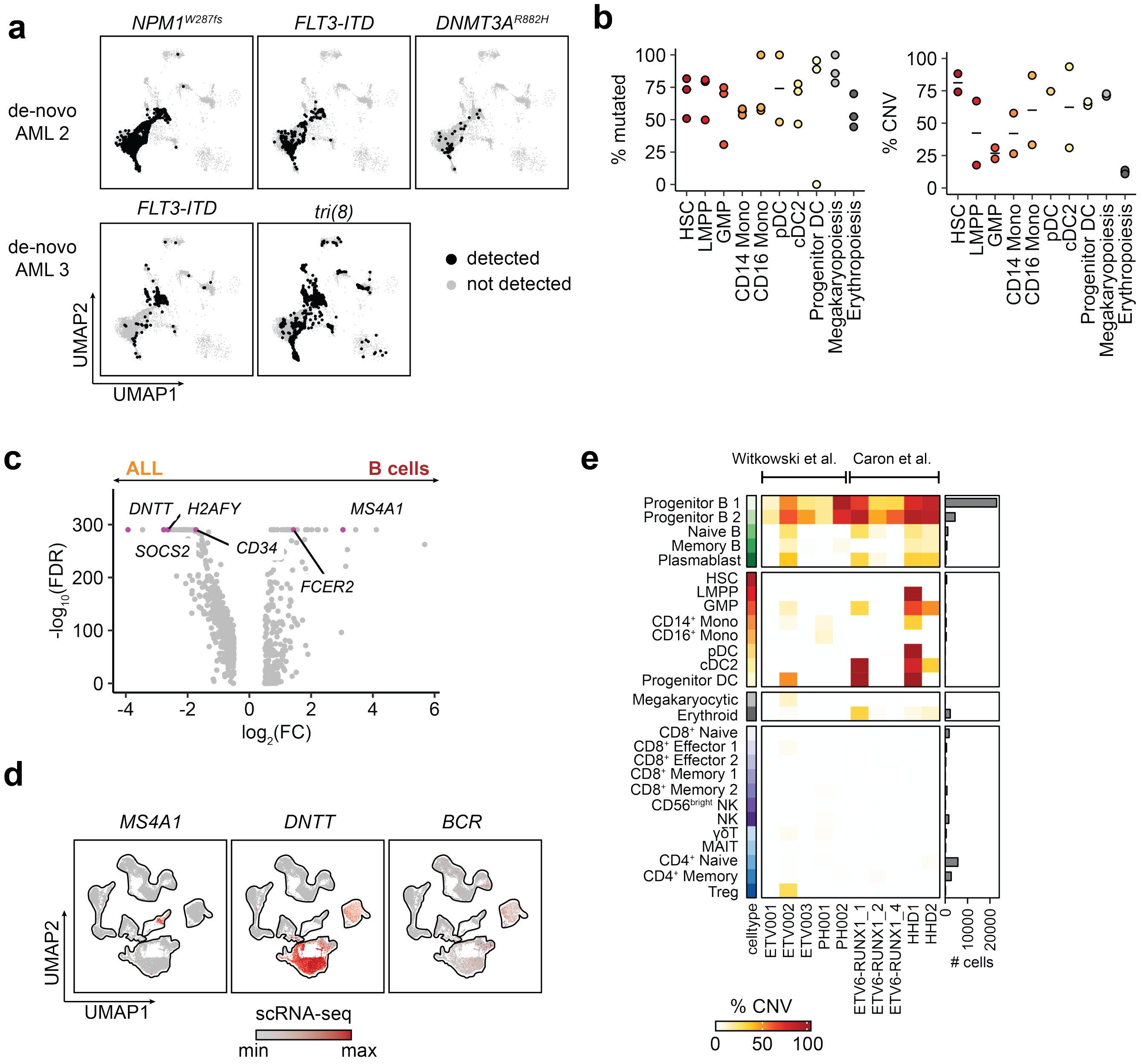

Supplementary Figure 12. Genotyping of de-novo AML and B ALL cells.

a UMAP projection demonstrating detection of somatic mutations and copy number changes in de-novo AML 2 and 3.

b Percentage of mutated cells across myeloid, megakaryocytic and erythroid lineage in de-novo AML 1-3 assessed with somatic mutations (left) or copy number changes (right).

c Differential gene expression analysis of *BCR::ABL1*⁻ B cells and *BCR::ABL1*⁺ ALL (acute lymphoblastic leukemia) cells.

d Expression of *MS4A1* (encoding CD20) and *DNTT* (Terminal deoxynucleotidyl transferase) in ALL bone marrow.

e Detection of CNV changes in re-analyzed data of B ALL single cell profiles by Wittkowski et al., Cancer Cell 2020[5] and Caron et al., Scientific Reports 2020[6].

ETV, ETV6/RUNX1 – ALL with *ETV6/RUNX1*, PH – ALL with Philadelphia chromosome (*BCR::ABL1*⁺), HHD – ALL with high hyper diploid karyotype

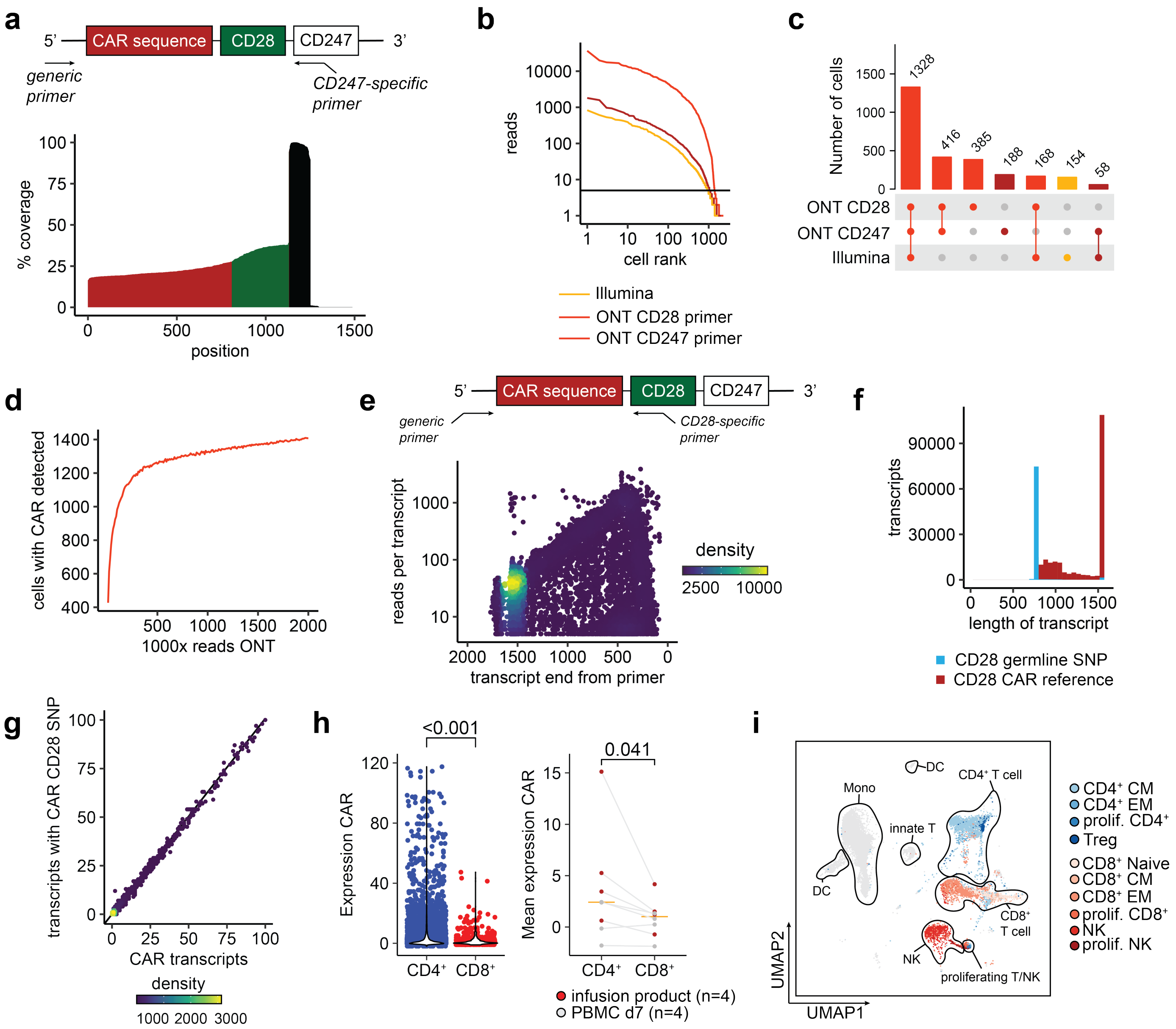

Supplementary Figure 13. Genotyping of CAR T cells with *nanoranger*.

a Coverage plot of Oxford Nanopore reads mapping to CAR sequence amplified with *CD247*-specific primer from cDNA of CAR T cell infusion product.

b Knee plot of cell barcodes with CAR T cell reads obtained from unamplified cDNA (Illumina) (yellow) and amplicons using a *CD28*- (light red) or *CD247*-specific primer (dark red).

c Number of cell barcodes detected from data shown in (b).

d, e Number of cells with detected CAR T cell transcript as a function of the number of analyzed reads (d) and amplification rate of reads as a function of their length (e). Shorter reads have higher amplification rate.

f Length distribution of transcripts containing the CD28 germline SNP (blue) versus the CD28 SNP encoded by the CAR expression vector (red).

g High correlation of identified CAR transcripts and transcripts containing the CAR-specific CD28 SNP per cell.

h Expression of CAR transcripts in CD4$^+$ and CD8$^+$ T cells from CAR T cell infusion products or circulating CAR T cells at day 7 after infusion shown for all cells (left) or as mean expression across samples (right).

i UMAP projection of single cell RNA sequencing profiles of CAR infusion products (n=4) and circulating immune cells at day 7 after CAR T cell infusion (n=4) annotated using reference map.

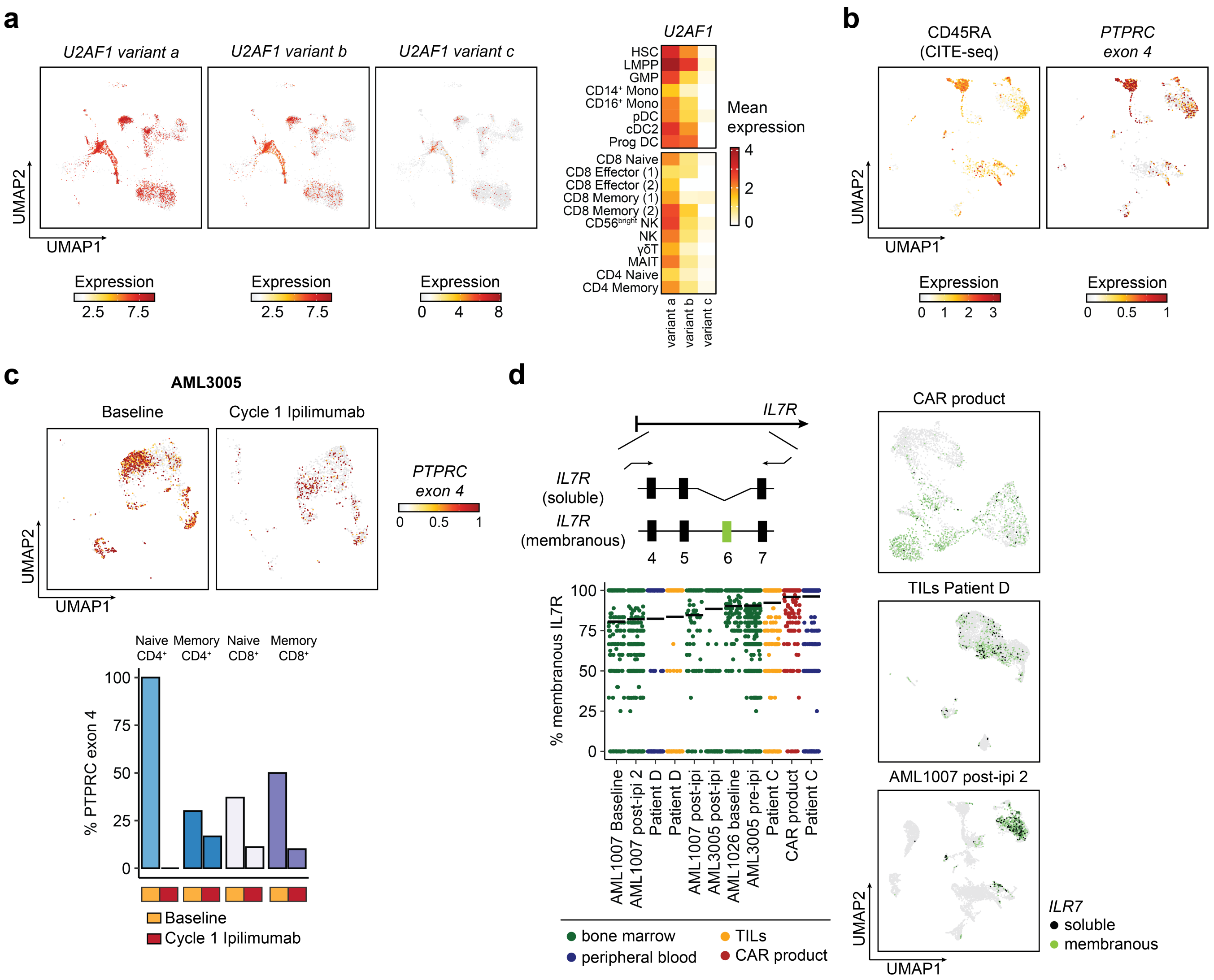

Supplementary Figure 14. Isoform detection in immune cells.
a UMAP projection of AML1002 single cell RNA sequencing profiles with detection of *U2AF1* variants a, b and c using *nanoranger* (left). Heatmap demonstrating expression of *U2AF1* variants a, b and c across cell types (right).
b Expression of CD45RA (CITE-seq, right) and *PTPRC* exon 4 (encoding CD45RA, left) in AML1007.
c Expression of *PTPRC* exon 4 (encoding CD45RA) in AML3005 before (UMAP left) and after infusion of ipilimumab (UMAP right). Percentage of cells with detectable exon 4 shown for T cell subpopulations in bar plot.
d Detection of soluble (black) and membranous (green) *IL7R* based on differential expression of exon 6 across different targeted Oxford Nanopore-sequenced single cell RNA sequencing libraries (left). Projection of both isoforms on UMAP of each library (right).

Supplementary Tables

Supplementary Table 1. Generic primers used for targeted amplification.

Name	Sequence	Comment
AAO272	CTACACGACGCTCTTCCGAT*C*T	standard venus
bio-AAO273	5Biosg/UAUAAGCAGTGGTATCAACGCAG*A*G	biotinylated mars PCR 1
rhCGA_venus	cgaCTACACGACGCTCTTCrCGATCc/3SpC3/	optimized venus primer for rhPCR PCR 2
CGAvenus.PS	CGACTACACGACGCTCT*T*C	optimized venus primer for nested PCR 3
mars.PS	AAGCAGTGGTATCAACGCAG*A*G	mars primer for whole cDNA library amplification
biotin	/5Biosg/	
spacer	/3SpC3/	
mars	AAGCAGTGGTATCAACGCAGAG	

Supplementary Table 2. Primers used for targeted amplification of fusion genes.

Name	Sequence	Comment
Bio_rh_BCRABL.1	/5Biosg/CACTGGGTCCAGCGAGAArGGTTTC/3SpC3/	BCR::ABL1 PCR2
mars_nested_BCRABL.3	AAGCAGTGGTATCAACGCAGAGAGAAGGTTTTCCTTGGAGTTCCAA	BCR::ABL1 PCR3
Bio_rh_RUNX1T1.1	/5Biosg/GAATGGCTCGTGCCATTAGTTAArCGTTGA/3SpC3/	RUNX1::RUNX1T1 PCR2
mars_nested_RUNX1T1.2	AAGCAGTGGTATCAACGCAGAGTCAGCCTAGATTGCGTCTTCA	RUNX1::RUNX1T1 PCR3
**Alternative primers**		
mars_nested_BCRABL.1	AAGCAGTGGTATCAACGCAGAGCTCAGACCCTGAGGCTCAA	used for testing only
mars_nested_BCRABL.2	AAGCAGTGGTATCAACGCAGAGAGCGAGAAGGTTTTCCTTGGA	used for testing only
mars_nested_BCRABL.4	AAGCAGTGGTATCAACGCAGAGGAGCGGCTTCACTCAGAC	used for testing only
mars_nested_BCRABL.5	AAGCAGTGGTATCAACGCAGAGAGGCTCAAAGTCAGATGCTACT	used for testing only
mars_nested_BCRABL.6	AAGCAGTGGTATCAACGCAGAGATGCTACTGGCCGCTGAA	used for testing only
mars_nested_RUNX1T1.1	AAGCAGTGGTATCAACGCAGAGTGGCATTGTTGGAGGAGTCA	used for testing only
mars_nested_RUNX1T1.3	AAGCAGTGGTATCAACGCAGAGCCACAGGTGAGTCTGGCAT	used for testing only
mars_nested_RUNX1T1.4	AAGCAGTGGTATCAACGCAGAGGCATTGTGGAGTGCTTCTCAGTA	used for testing only

Supplementary Table 3. Primers used for targeted amplification of CAR transcripts.

**Name**	**Sequence**	**Comment**
Bio_rh_CD28.1	/5Biosg/CAGGAGCCTGCTCCTCTTArCTCCTA/3SpC3/	CD28 primer PCR 2
mars_nested_CD28.1	AAGCAGTGGTATCAACGCAGAGTCCTCTTACTCCTCACCCAGAA	CD28 primer PCR 3
Bio_rh_CD247.1	/5Biosg/TCATTGTACAGGCCTTCCTGArGGGTTA/3SpC3/	CD247 primer PCR 2
mars_nested_CD247.1	AAGCAGTGGTATCAACGCAGAGGGCCACGTCTCTTGTCCAA	CD247 primer PCR 3
**Alternative primers**		
mars_nested_CD28.2	AAGCAGTGGTATCAACGCAGAGGGCCACTGTTACTAGCAAGCTATA	used for testing only
mars_nested_CD28.3	AAGCAGTGGTATCAACGCAGAGAGCACCCAAAAGGGCTTAGAA	used for testing only
mars_nested_CD28.4	AAGCAGTGGTATCAACGCAGAGAGGTGTTTCCCTTTCACATGGATA	used for testing only
mars_nested_CD247.2	AAGCAGTGGTATCAACGCAGAGTCGTACTCCTCTCTTCGTCCTA	used for testing only
mars_nested_CD247.3	AAGCAGTGGTATCAACGCAGAGTTGAGCTCGTTATAGAGCTGGTT	used for testing only
mars_nested_CD247.4	AAGCAGTGGTATCAACGCAGAGCGCTCCTGCTGAACTTCA	used for testing only

Supplementary Table 4. Primers used for targeted amplification of somatic mutations associated with AML/MDS.

Name	Sequence	Comment
Bio_rh_ASXL1.1	/5Biosg/CAAGCTACCCTGCAGCAArCTGCAC/3SpC3/	
mars_nested_ASXL1.1	AAGCAGTGGTATCAACGCAGAGCTGGCCAGCAGTAGGGAA	
Bio_rh_ASXL1.2	/5Biosg/CAGTTCTAACATCCGGATGCAArCTGAGA/3SpC3/	
mars_nested_ASXL1.2	AAGCAGTGGTATCAACGCAGAGGTCCCCAGTGGGAGCAA	
Bio_rh_DNMT3A.2	/5Biosg/GTCCCTTACACACACGCAArAATACC/3SpC3/	
mars_nested_DNMT3A.1	AAGCAGTGGTATCAACGCAGAGATGACTGGCACGCTCCAT	
Bio_rh_DNMT3A.3	/5Biosg/CCTCAGGTTCCACCCACrATGTCA/3SpC3/	
mars_nested_DNMT3A.2	AAGCAGTGGTATCAACGCAGAGGGGCCAGAAGGCTGGAA	
Bio_rh_FLT3-ITD.1	/5Biosg/GACATGAGTGCCTCTCTTTCArGAGCTa/3SpC3/	
Bio_rh_FLT3-ITD.2	/5Biosg/CCAAAAGCACCTGATCCTAGTACrCTTCCa/3SpC3/	
mars_nested_FLT3-ITD.1	AAGCAGTGGTATCAACGCAGAGCTTTCAGCATTTTGACGGCAACC	
mars_nested_FLT3-ITD.2	AAGCAGTGGTATCAACGCAGAGGCACCTGATCCTAGTACCTTCC	
Bio_rh_FLT3-TKD.1	/5Biosg/GGCCATCCATTTTACAGGCArGACGGt/3SpC3/	
mars_nested_FLT3-TKD.1	AAGCAGTGGTATCAACGCAGAGCCCCTGACAACATAGTTGGAATCA	
Bio_rh_IDH2.1	/5Biosg/TCTCCAGCATCTGGGCAArACCTGC/3SpC3/	
mars_nested_IDH2.1	AAGCAGTGGTATCAACGCAGAGCCTGAGCCACCATGTCATCAA	
Bio_rh_NPM1.1	/5Biosg/CGGTAGGGAAAGTTCTCACTCTGCrTAATAc/3SpC3/	
mars_nested_NPM1.1	AAGCAGTGGTATCAACGCAGAGGGACAGCCAGATATCAACTGTTACAG	
Bio_rh_NRAS.1	/5Biosg/GGTGTGTTTGTGCTGTGGAArGAACCA/3SpC3/	
mars_nested_NRAS.1	AAGCAGTGGTATCAACGCAGAGGTTACATCACCACACATGGCAA	
Bio_rh_RUNX1.1	/5Biosg/CGCCCACCATGGAGAArCTGGTC/3SpC3/	
Bio_rh_RUNX1.2	/5Biosg/ACTGTGATTTTGATGGCTCTGTGrGTAGGA/3SpC3/	
mars_nested_RUNX1.1	AAGCAGTGGTATCAACGCAGAGGGTGAAGGCGCCTGGATA	
mars_nested_RUNX1.2	AAGCAGTGGTATCAACGCAGAGCTTGCGGTGGGTTTGTGAA	
Bio_rh_SF3B1.1	/5Biosg/CATCCTGTGCTGCCAGAArGTGTTC/3SpC3/	
Bio_rh_SF3B1.2	/5Biosg/GAGTTGCTGCTTCAGCCArAGGCAT/3SpC3/	
mars_nested_SF3B1.1	AAGCAGTGGTATCAACGCAGAGACTGTTTTACCACCTTCAGCACAA	
Bio_rh_SRSF2.1	/5Biosg/GAAGTTTGCCAACTGAGGCAArAGCTTC/3SpC3/	
mars_nested_SRSF2.1	AAGCAGTGGTATCAACGCAGAGAGGTCGACCGAGATCGAGAA	
Bio_rh_STAG2.1	/5Biosg/CTCTCTTCGGAGTGACATCTGAArAGGTCC/3SpC3/	
mars_nested_STAG2.1	AAGCAGTGGTATCAACGCAGAGGCCAAATTTAAAGGTGGATGGCTCT	
Bio_rh_STAG2.2	/5Biosg/GGCATCACTATACATCTTCATCCArAATGCA/3SpC3/	
mars_nested_STAG2.2	AAGCAGTGGTATCAACGCAGAGTCGCATCACGGTATCTATGTACAA	
Bio_rh_TET2.1	/5Biosg/CTCATTCATGCTCTTATGCTGGTAArAAGACT/3SpC3/	
mars_nested_TET2.1	AAGCAGTGGTATCAACGCAGAGGTTGTGGCATGCAGCTCA	
Bio_rh_TET2.2	/5Biosg/CTGCATGACTGGTCCTGAArAGTCGA/3SpC3/	
mars_nested_TET2.2	AAGCAGTGGTATCAACGCAGAGAGTCGCAAAAGTTCTGCCAAC	
Bio_rh_TET2.4	/5Biosg/TGCTGGCAGTTGTCCTGTArGCTCTA/3SpC3/	
mars_nested_TET2.3	AAGCAGTGGTATCAACGCAGAGGGTTATGCTTGAGGTGTTCTGACA	
Bio_rh_TP53.1	/5Biosg/TGGGCAGTGCTCGCTTArGTGCTA/3SpC3/	
mars_nested_TP53.1	AAGCAGTGGTATCAACGCAGAGTCCCCTTTCTTGCGGAGATT	
Bio_rh_U2AF1.1	/5Biosg/CCTTATGAACTGGTTTGGTCAATCAArCTACAC/3SpC3/	
mars_nested_U2AF1.1	AAGCAGTGGTATCAACGCAGAGGCTCTCTGGAAATGGGCTTCAA	
**Alternative primers**		
Bio_rh_DNMT3A.1	/5Biosg/TCCTTCAGCGGAGCGAArGAGGTA/3SpC3/	Not used
Bio_rh_PTPN11.1	/5Biosg/AGGGTGGCGTTGGAGTArCAAGGA/3SpC3/	Not used
mars_nested_PTPN11.1	AAGCAGTGGTATCAACGCAGAGTCCTGCGCTGTAGTGTTTCAA	Not used
Bio_rh_TET2.3	/5Biosg/CATGTATGGATTGGTGGATCCAGArAGCAGT/3SpC3/	Not used

Supplementary Table 5. Primers used for targeted amplification of mitochondrial transcripts.

Name	Sequence	Comment
Bio_rh_mars_ND1.1	/5Biosg/AAGCAGTGGTATCAACGCAGAGGGGAATGCTGGAGATTGTArATGGGA/3SpC3/	PCR 2
mars_nested_ND1.1	AAGCAGTGGTATCAACGCAGAGGGAGATTGTAATGGGTATGGAGACATA	PCR 3
Bio_rh_mars_ND4.1	/5Biosg/AAGCAGTGGTATCAACGCAGAGAGAGGAAAACCCGGTAATGATrGTCGGA/3SpC3/	PCR 2
mars_nested_ND4.1	AAGCAGTGGTATCAACGCAGAGGATGTCGGGGTTGAGGGATA	PCR 3
Bio_rh_mars_ND5.2	/5Biosg/AAGCAGTGGTATCAACGCAGAGGTTTTGGCTCGTAAGAAGGCrCTAGAC/3SpC3/	PCR 2
mars_nested_ND5.2	AAGCAGTGGTATCAACGCAGAGTGTGCGGTGTGTGATGCTA	PCR 3
rh_mars_ATP6	AAGCAGTGGTATCAACGCAGAGTTATGTGTTGTCGTGCAGGTrAGAGGA/3SpC3/	PCR 3 only
rh_mars_COX1.2	AAGCAGTGGTATCAACGCAGAGGGCATCCATATAGTCACTCCArGGTTTC/3SpC3/	PCR 3 only
rh_mars_COX1.4	AAGCAGTGGTATCAACGCAGAGGATGAGTTTGCTAATACAATGCCArGTCAGA/3SpC3/	PCR 3 only
rh_mars_COX2	AAGCAGTGGTATCAACGCAGAGGGTAAATACGGGCCCTATTTCrAAAGAC/3SpC3/	PCR 3 only
rh_mars_COX3	AAGCAGTGGTATCAACGCAGAGCCCTCATCAATAGATGGAGACATArCAGAAC/3SpC3/	PCR 3 only
rh_mars_CYTB	AAGCAGTGGTATCAACGCAGAGGGCCCATTTGAGTATTTTGTTTTCArATTAGA/3SpC3/	PCR 3 only
rh_mars_ND1.4	AAGCAGTGGTATCAACGCAGAGGATAGTAGAATGATGGCTAGGGTGrACTTCC/3SpC3/	PCR 3 only
rh_mars_ND2	AAGCAGTGGTATCAACGCAGAGGGGAGATAGGTAGGAGTAGCrGTGGTC/3SpC3/	PCR 3 only
rh_mars_ND3	AAGCAGTGGTATCAACGCAGAGTCAGTCTAATCCTTTTTGTAGTCACTrCATAGA/3SpC3/	PCR 3 only
rh_mars_ND4.6	AAGCAGTGGTATCAACGCAGAGGGAAGGGAGCCTACTAGGrGTGTAA/3SpC3/	PCR 3 only
rh_mars_ND5.4	AAGCAGTGGTATCAACGCAGAGGAGTAATAGATAGGGCTCAGGCrGTTTGC/3SpC3/	PCR 3 only
rh_mars_ND5.6	AAGCAGTGGTATCAACGCAGAGAGTGGTGATAGCGCCTAAGrCATAGC/3SpC3/	PCR 3 only
rh_mars_ND5.8	AAGCAGTGGTATCAACGCAGAGGAAGGATATAATTCCTACGCCCTrCTCAGA/3SpC3/	PCR 3 only
rh_mars_ND6	AAGCAGTGGTATCAACGCAGAGCTATTCCCCCGAGCAATCTrCAATTC/3SpC3/	PCR 3 only
rh_mars_RNR1	AAGCAGTGGTATCAACGCAGAGCGTCCAAGTGCACTTTCCrAGTACC/3SpC3/	PCR 3 only
rh_mars_RNR2.2	AAGCAGTGGTATCAACGCAGAGGAAGGCGCTTTGTGAAGTAGrGCCTTC/3SpC3/	PCR 3 only
rh_mars_RNR2.5	AAGCAGTGGTATCAACGCAGAGGCCTCTAATACTGGTGATGCTrAGAGGA/3SpC3/	PCR 3 only

Supplementary Table 6. Primers used for targeted amplification of transcripts with alternative splicing (isoforms).

Name	Sequence	Comment
Bio_rh_CTLA4.Ex4.1	/5Biosg/CCCAAATGCACAAACAAATAAAAATAGCTGrGATAGC/3SpC3/	PCR 2
mars_nested_CTLA4.Ex4.1	AAGCAGTGGTATCAACGCAGAGGCTTTTCACATTCTGGCTCTGTT	PCR 3
Bio_rh_IL7R.Ex7.1	/5Biosg/CCTTCCACTTCATCTCTAGCTTGArATGTCT/3SpC3/	PCR 2
mars_nested_IL7R.Ex7.1	AAGCAGTGGTATCAACGCAGAGTGAATCTGGCAGTCCAGGAA	PCR 3
Bio_rh_PTPRC.Ex7.1	/5Biosg/TCATCACATGTTGGCTTAGATGGArGTAGTC/3SpC3/	PCR 2
mars_nested_PTPRC.Ex7.1	AAGCAGTGGTATCAACGCAGAGGAAATGACAGCGCTTCCAGAA	PCR 3
**Other primers**		
Bio_rh_BCL2L11.Ex6.1	/5Biosg/TCTCGGCTCCGCAAAGAArCCTGTA/3SpC3/	Not shown
Bio_rh_BCL2L11.tr.Ex6.1	/5Biosg/TGCTCTTGAGGTAAAGTGACTTCAArAGCTGA/3SpC3/	Not shown
Bio_rh_CD6.Ex6.1	/5Biosg/TCTATAGTGACTGTCTGAACACTTGCrAGGGAA/3SpC3/	Not shown
Bio_rh_CD6.Ex10.1	/5Biosg/CAACTCTTCAAGTCCTTCCTCCArAGGGTA/3SpC3/	Not shown
Bio_rh_CD8B.Ex7.1	/5Biosg/TCAGGATCCATGGGTTAAGCArGCTTCC/3SpC3/	Not shown
Bio_rh_CD44.Ex16.1	/5Biosg/GGGATGCCAAGATGATCAGCrCATTCC/3SpC3/	Not shown
Bio_rh_FOXP3.Ex3.1	/5Biosg/GTTGAGAGCTGGTGCATGAArATGTGA/3SpC3/	Not shown
Bio_rh_FOXP3.Ex8.1	/5Biosg/TCGGATGATGCCACAGATGAArGCCTTA/3SpC3/	Not shown
Bio_rh_IKZF1.Ex9.1	/5Biosg/CCCCAGGTAGTTGATGGCrGTTGTC/3SpC3/	Not shown
Bio_rh_MALT1.Ex8.1	/5Biosg/ACATCCACCAAAGGAGCTTTGArGCTTGA/3SpC3/	Not shown
Bio_rh_SLAMF6.Ex3.1	/5Biosg/GAGACATTGTCATCTGCATCCTCrCACAGT/3SpC3/	Not shown
mars_nested_BCL2L11.Ex6.1	AAGCAGTGGTATCAACGCAGAGCCAGGCGGACAATGTAACGTAA	Not shown
mars_nested_BCL2L11.tr.Ex6.1	AAGCAGTGGTATCAACGCAGAGGCAAAAGCTCCAATCCTGCAA	Not shown
mars_nested_CD6.Ex6.1	AAGCAGTGGTATCAACGCAGAGGGGAGTGGACAGATTGTGCAA	Not shown
mars_nested_CD6.Ex10.1	AAGCAGTGGTATCAACGCAGAGTGGAACCTGCTTTGCTGGA	Not shown
mars_nested_CD8B.Ex7.1	AAGCAGTGGTATCAACGCAGAGGAGGTTGTAGTATTGCTGTAGTATCCA	Not shown
mars_nested_CD44.Ex16.1	AAGCAGTGGTATCAACGCAGAGGTGTCCTTATAGGACCAGAGGTT	Not shown
mars_nested_FOXP3.Ex8.1	AAGCAGTGGTATCAACGCAGAGGGTCAGTGCCATTTTCCCA	Not shown
mars_nested_FOXP3.Ex3.1	AAGCAGTGGTATCAACGCAGAGCCTGGAGGAGTGCCTGTAA	Not shown
mars_nested_IKZF1.Ex9.1	AAGCAGTGGTATCAACGCAGAGTGTTGATGGCTTGGTCCATCA	Not shown
mars_nested_MALT1.Ex8.1	AAGCAGTGGTATCAACGCAGAGTCGCCAAAGGCTGGTCA	Not shown
mars_nested_SLAMF6.Ex3.1	AAGCAGTGGTATCAACGCAGAGAGCAAGTCAGATGGAGCTCA	Not shown

Supplementary Table 7. Primers used for comparison of *nanoranger* with genotyping of transcriptomes (GoT).

Name	Sequence
DNMT3A_RT	AGCAAGTGAGAAGCATCGTGTCTGTTTAACTTTGTGTCGCTACCTCA
RUNX1_RT	AGCAAGTGAGAAGCATCGTGTCTGTGATTTTGATGGCTCTGTGGTA
SF3B1_RT	AGCAAGTGAGAAGCATCGTGTCCTTCAGCCAAGGCAGCAA
Additive Primer	AGCAAGTGAGAAGCATCGTG*T*C
Bio_Additive	/5Biosg/AGCAAGTGAGAAGCATCGTG*T*C
**GoT PCR1 Primers**	
**Name**	**Sequence**
DNMT3A_Staggered0	CACCCGAGAATTCCAATGACTGGCACGCTCCAT
DNMT3A_Staggered1	CACCCGAGAATTCCAAATGACTGGCACGCTCCAT
DNMT3A_Staggered2	CACCCGAGAATTCCATTATGACTGGCACGCTCCAT
DNMT3A_Staggered3	CACCCGAGAATTCCACATATGACTGGCACGCTCCAT
RUNX1_Staggered0	CACCCGAGAATTCCACTTGCGGTGGGTTTGTGAA
RUNX1_Staggered1	CACCCGAGAATTCCAACTTGCGGTGGGTTTGTGAA
RUNX1_Staggered2	CACCCGAGAATTCCATTCTTGCGGTGGGTTTGTGAA
RUNX1_Staggered3	CACCCGAGAATTCCACATCTTGCGGTGGGTTTGTGAA
SF3B1_Staggered0	CACCCGAGAATTCCACAAAGCACTGATGGTCCGAA
SF3B1_Staggered1	CACCCGAGAATTCCAACAAAGCACTGATGGTCCGAA
SF3B1_Staggered2	CACCCGAGAATTCCATTCAAAGCACTGATGGTCCGAA
SF3B1_Staggered3	CACCCGAGAATTCCACATCAAAGCACTGATGGTCCGAA
10x 5'kit bead oligo	CTACACGACGCTCTTCCGATCT-NNNNNNNNNNNNNNNN-NNNNNNNNNN-TTTCTTATATrGrGrG
SI-PCR	AATGATACGGCGACCACCGAGATCTACACTCTTTCCCTACACGACGCTC
P5	AATGATACGGCGACCACCGAGATCTACAC
RPI-X	CAAGCAGAAGACGGCATACGAGAT*XXXXXXXX*GTGACTGGAGTTCCTTGGCACCCGAGAATTCCA
P7	CAAGCAGAAGACGGCATACGAGAT

Supplementary Table 8. Antibodies used for FACS sort of tumor-infiltrating lymphocytes.

Antigen	Fluorochrome	Clone	Vendor	Cat. no	Dilution
anti-human CD45	PE-Cy7	2D1	BioLegend	368532	1:50
anti-human CD3	APC-Cy7	UCHT1	BioLegend	300426	1:50
live/dead	Zombie Aqua fixable viability kit	n/a	BioLegend	423102	1:400

Supplementary Table 9. Number of cells bearing somatic nuclear mutations across cell types (I).

	HSC		LMPP		GMP		CD14+ Mono		CD16+ Mono	
	mutated	cells	mutated	cells	mutated	cells	mutated	cells	mutated	cells
AML1002	182	280	120	172	5	8	21	44	1	30
AML1007	62	83	31	43	31	64	151	325	5	42
AML1010	126	231	32	56	21	48	4	15	2	4
AML1019	54	86	2	14	0	2	0	1	NA	NA
AML1022	202	401	208	357	17	29	267	522	1	6
AML1026	93	141	18	27	15	23	0	2	NA	NA
AML3003	18	44	34	58	5	15	0	1	NA	NA
AML3005	163	275	114	145	8	17	NA	NA	0	5
AML8007	85	116	110	123	14	17	NA	NA	NA	NA
	cDC2		pDC		Prog_DC		Megakaryopoiesis		Erythropoiesis	
	mutated	cells	mutated	cells	mutated	cells	mutated	cells	mutated	cells
AML1002	3	7	2	3	2	8	208	388	119	186
AML1007	36	68	0	2	10	14	8	19	63	157
AML1010	1	1	NA	NA	2	3	1	1	19	38
AML1019	NA	NA	NA	NA	NA	NA	4	7	1	6
AML1022	40	79	NA	NA	4	4	79	138	67	104
AML1026	NA	NA	NA	NA	0	2	12	18	59	89
AML3003	0	2	8	18	NA	NA	1	3	3	48
AML3005	1	2	3	6	3	14	3	23	23	834
AML8007	NA	NA	NA	NA	1	4	60	74	137	187
	CD4+ Naïve		CD4+ Memory		Treg		CD8+ Naive		CD8+ Effector 1	
	mutated	cells	mutated	cells	mutated	cells	mutated	cells	mutated	cells
AML1002	5	208	7	623	0	22	0	29	3	249
AML1007	0	16	5	623	0	36	1	22	0	5
AML1010	NA	NA	NA	NA	NA	NA	NA	NA	0	2
AML1019	0	1	0	28	0	2	NA	NA	0	4
AML1022	1	4	0	12	NA	NA	0	1	NA	NA
AML1026	1	2	1	115	0	4	2	14	0	2
AML3003	0	16	0	107	0	6	0	5	0	9
AML3005	2	824	11	4515	0	237	0	148	0	120
AML8007	0	10	5	102	0	2	0	16	0	17

Supplementary Table 10. Number of cells bearing somatic nuclear mutations across cell types (II).

	$CD8^+$ Effector 2		$CD8^+$ Memory 1		$CD8^+$ Memory 2		$CD56^{bright}$ NK		NK	
	mutated	cells	mutated	cells	mutated	cells	mutated	cells	mutated	cells
AML1002	0	6	0	9	3	516	2	7	52	871
AML1007	0	1	0	1	1	83	1	15	4	230
AML1010	0	1	NA	NA	0	6	NA	NA	0	4
AML1019	0	2	NA	NA	0	8	0	1	0	35
AML1022	NA	NA	NA	NA	0	5	0	3	0	21
AML1026	NA	NA	NA	NA	3	124	0	1	1	45
AML3003	0	4	NA	NA	0	15	1	5	10	146
AML3005	0	49	0	14	0	151	1	29	1	493
AML8007	1	14	NA	NA	0	40	0	2	4	42
	**gdT**		**MAIT**		**Progenitor B 1**		**Progenitor B 2**		**Naïve B**	
	mutated	cells	mutated	cells	mutated	cells	mutated	cells	mutated	cells
AML1002	0	1	0	10	37	62	3	3	0	10
AML1007	NA	NA	1	28	1	2	NA	NA	NA	NA
AML1010	NA	NA	NA	NA	NA	NA	NA	NA	NA	NA
AML1019	NA	NA	0	1	NA	NA	NA	NA	0	13
AML1022	NA	NA	NA	NA	1	6	NA	NA	NA	NA
AML1026	NA	NA	0	1	NA	NA	NA	NA	NA	NA
AML3003	1	3	0	9	5	12	NA	NA	NA	NA
AML3005	0	6	0	71	84	150	4	5	0	14
AML8007	0	1	0	2	2	4	0	1	0	3
	**Memory B**		**Plasmablast**							
	mutated	cells	mutated	cells						
AML1002	0	20	0	11						
AML1007	NA	NA	0	2						
AML1010	NA	NA	NA	NA						
AML1019	0	8	NA	NA						
AML1022	0	1	NA	NA						
AML1026	NA	NA	NA	NA						
AML3003	NA	NA	NA	NA						
AML3005	0	12	0	28						
AML8007	0	6	0	5						

Supplementary references

1. Oliveira, G., Stromhaug, K., Klaeger, S., Kula, T., Frederick, D.T., Le, P.M., Forman, J., Huang, T., Li, S., Zhang, W., et al. (2021). Phenotype, specificity and avidity of antitumour CD8+ T cells in melanoma. Nature *596*, 119–125. 10.1038/s41586-021-03704-y.

2. van Galen, P., Hovestadt, V., Wadsworth Ii, M.H., Hughes, T.K., Griffin, G.K., Battaglia, S., Verga, J.A., Stephansky, J., Pastika, T.J., Lombardi Story, J., et al. (2019). Single-Cell RNA-Seq Reveals AML Hierarchies Relevant to Disease Progression and Immunity. Cell *176*, 1265-1281.e24. 10.1016/j.cell.2019.01.031.

3. Bottomly, D., Long, N., Schultz, A.R., Kurtz, S.E., Tognon, C.E., Johnson, K., Abel, M., Agarwal, A., Avaylon, S., Benton, E., et al. (2022). Integrative analysis of drug response and clinical outcome in acute myeloid leukemia. Cancer Cell *40*, 850-864.e9. 10.1016/j.ccell.2022.07.002.

4. Heaton, H., Talman, A.M., Knights, A., Imaz, M., Gaffney, D.J., Durbin, R., Hemberg, M., and Lawniczak, M.K.N. (2020). Souporcell: robust clustering of single-cell RNA-seq data by genotype without reference genotypes. Nat Methods *17*, 615–620. 10.1038/s41592-020-0820-1.

5. Witkowski, M.T., Dolgalev, I., Evensen, N.A., Ma, C., Chambers, T., Roberts, K.G., Sreeram, S., Dai, Y., Tikhonova, A.N., Lasry, A., et al. (2020). Extensive Remodeling of the Immune Microenvironment in B Cell Acute Lymphoblastic Leukemia. Cancer Cell *37*, 867-882.e12. 10.1016/j.ccell.2020.04.015.

6. Caron, M., St-Onge, P., Sontag, T., Wang, Y.C., Richer, C., Ragoussis, I., Sinnett, D., and Bourque, G. (2020). Single-cell analysis of childhood leukemia reveals a link between developmental states and ribosomal protein expression as a source of intra-individual heterogeneity. Sci Rep *10*, 8079. 10.1038/s41598-020-64929-x.
