# Supplementary material for: Integrative genotyping of cancer and immune phenotypes by long-read sequencing
Source: Nat Commun. 2024 Jan 2;15:32. doi: 10.1038/s41467-023-44137-7 (PMC10762175; doi:10.1038/s41467-023-44137-7)
Supplement: Supplementary file 3 — Reporting Summary [file 41467_2023_44137_MOESM3_ESM.pdf]

nature portfolio

Corresponding author(s): Catherine J. Wu

Last updated by author(s): Nov 1, 2023

Reporting Summary

Nature Portfolio wishes to improve the reproducibility of the work that we publish. This form provides structure for consistency and transparency in reporting. For further information on Nature Portfolio policies, see our Editorial Policies and the Editorial Policy Checklist.

Statistics

For all statistical analyses, confirm that the following items are present in the figure legend, table legend, main text, or Methods section.

n/a	Confirmed	
☐	☒	The exact sample size (*n*) for each experimental group/condition, given as a discrete number and unit of measurement
☐	☒	A statement on whether measurements were taken from distinct samples or whether the same sample was measured repeatedly
☐	☒	The statistical test(s) used AND whether they are one- or two-sided<br>*Only common tests should be described solely by name; describe more complex techniques in the Methods section.*
☒	☐	A description of all covariates tested
☐	☒	A description of any assumptions or corrections, such as tests of normality and adjustment for multiple comparisons
☐	☒	A full description of the statistical parameters including central tendency (e.g. means) or other basic estimates (e.g. regression coefficient) AND variation (e.g. standard deviation) or associated estimates of uncertainty (e.g. confidence intervals)
☐	☒	For null hypothesis testing, the test statistic (e.g. *F*, *t*, *r*) with confidence intervals, effect sizes, degrees of freedom and *P* value noted<br>*Give P values as exact values whenever suitable.*
☒	☐	For Bayesian analysis, information on the choice of priors and Markov chain Monte Carlo settings
☒	☐	For hierarchical and complex designs, identification of the appropriate level for tests and full reporting of outcomes
☒	☐	Estimates of effect sizes (e.g. Cohen's *d*, Pearson's *r*), indicating how they were calculated

Our web collection on statistics for biologists contains articles on many of the points above.

Software and code

Policy information about availability of computer code

Data collection	raw scRNA-seq data were processed with cellranger/7.0.0; raw scATAC-seq data were processed with cellranger-atac/2.0.0; mitochondrial DNA mutation calling was performed with mgatk/0.6.1 for scATAC-seq data and maegatk for scRNA-seq/targeted ONT data; raw LSK112/114 Q20+ Oxford Nanopore reads from MinION and GridION were processed using the guppy basecaller; data from NCBI GEO was either downloaded directly or using SRA Toolkit/2.10.7
Data analysis	raw sequences reads were visualized using IGV/2.14.1; Oxford Nanopore data were processed using the nanoranger pipeline (https://github.com/mehdiborji/nanoranger) which is written in python3 using pysam; sequence alignments are performed with minimap2 and STAR; sam/bam file handling using samtools; raw T cell receptor sequences were analyzed using MiXCR (https://github.com/milaboratory/mixcr); downstream processing including variant calling was performed using R scripts (https://github.com/liviuspenter/nanoranger.R); deconcatenation of MAS-ISO-seq data was performed using longbow (https://github.com/broadinstitute/longbow); sequencing coverage was calculated using bedtools; copy number variants from scRNA-seq profiles were called using numbat (https://github.com/kharchenkolab/numbat); analyses and visualization of processed sequencing data were performed in RStudio 2022.07.1+554 using R 4.2.1; major R packages include Seurat/4.3.0, ggplot2/3.4.0, dplyr/1.0.10<br>The custom code for reproduction of figures can be found under https://github.com/liviuspenter/ONT-lineage-tracing.

For manuscripts utilizing custom algorithms or software that are central to the research but not yet described in published literature, software must be made available to editors and reviewers. We strongly encourage code deposition in a community repository (e.g. GitHub). See the Nature Portfolio guidelines for submitting code & software for further information.

Data

Policy information about availability of data

All manuscripts must include a data availability statement. This statement should provide the following information, where applicable:

- Accession codes, unique identifiers, or web links for publicly available datasets
- A description of any restrictions on data availability
- For clinical datasets or third party data, please ensure that the statement adheres to our policy

Gene expression matrices of samples sequenced for this work are deposited with the NCBI Gene Expression Omnibus (GEO) (accession number GSE243227 [https://www.ncbi.nlm.nih.gov/geo/query/acc.cgi?acc= GSE243227]).
The raw long-read sequencing data generated for this work are available on the NCBI sequencing read archive (SRA) (project number PRJNA935418 [https://www.ncbi.nlm.nih.gov/bioproject/PRJNA935418]).
Previously published gene expression matrices for samples from ETCTN/CTEP 10026 are available on NCBI GEO (accession GSE223844 [https://www.ncbi.nlm.nih.gov/geo/query/acc.cgi?acc=GSE223844]). Additional previously generated single cell RNA Illumina raw sequencing data are available on NCBI's Database of Genotypes and Phenotypes (dbGaP; https://www.ncbi.nlm.nih.gov/gap) under accession number phs003015.v1
[https://www.ncbi.nlm.nih.gov/projects/gap/cgi-bin/study.cgi?study_id=phs003015.v1.p1] (AML), or under accession number phs001451.v4.p1
[https://www.ncbi.nlm.nih.gov/projects/gap/cgi-bin/study.cgi?study_id=phs001451.v4.p1] (melanoma) and phs002922.v1.p1
[https://www.ncbi.nlm.nih.gov/projects/gap/cgi-bin/study.cgi?study_id=phs002922.v1.p1] (CAR).

Human research participants

Policy information about studies involving human research participants and Sex and Gender in Research.

Reporting on sex and gender	Sex or gender were not considered for the reported research as all methods and analyses utilized in the research are agnostic of sex or gender, are not influenced by them and equally apply to any sex or gender.
Population characteristics	Studied samples were obtained from patients with acute myeloid leukemia (AML), acute lymphoblastic leukemia (ALL), large B cell lymphoma or melanoma. For more information on AML patients, please see Tables 1 and 2.
Recruitment	Samples were obtained from participants in clinical trials ETCTN/CTEP 10026 (NCT02890329), NCT01970358 and as part of IRB-approved protocols at Dana-Farber/Harvard Cancer Center (DFHCC #16-206; #17-561; #17-718).
Ethics oversight	The study protocols were approved by institutional review boards at participating centers including Dana-Farber Cancer Center (Boston, MA USA) and Massachusetts General Hospital (Boston, MA USA).

Note that full information on the approval of the study protocol must also be provided in the manuscript.

Field-specific reporting

Please select the one below that is the best fit for your research. If you are not sure, read the appropriate sections before making your selection.

☒ Life sciences ☐ Behavioural & social sciences ☐ Ecological, evolutionary & environmental sciences

For a reference copy of the document with all sections, see nature.com/documents/nr-reporting-summary-flat.pdf

Life sciences study design

All studies must disclose on these points even when the disclosure is negative.

Sample size	No predetermined sample size was utilized. Sample sizes were chosen to demonstrate reproducibility and feasibility (mixing experiment with K562/Kasumi1: 4 conditions; somatic mutations in AML: 9 patients with 13 samples for secondary AML and 3 patients with 3 samples for de-novo AML; tracking of CAR sequences with 8 samples; T cell isoform detection in 8 samples; identification of BCR::ABL1 in 4 ALL cases; head-to-head comparison with GoT in one selected case).
Data exclusions	No samples were excluded from this study.
Replication	Where possible public data was used for orthogonal validation including data from van Galen et al., Cell 2019; Witkowski et al., Cancer Cell 2020 and Caron et al., Scientific Reports 2020. Single cell libraries were not replicated (not feasible due to costs), but individual cells of the same population can be considered biological replicates within the same library.
Randomization	Randomization was not relevant for this research. Study samples were obtained from non-randomized study participants of phase I clinical trials (NCT02890329 and NCT01970358) or observational studies of response.

Blinding	Blinding was not relevant for this research. Study samples were obtained from non-blinded phase I clinical trials (NCT02890329 and NCT01970358) or observational studies of response.

Reporting for specific materials, systems and methods

We require information from authors about some types of materials, experimental systems and methods used in many studies. Here, indicate whether each material, system or method listed is relevant to your study. If you are not sure if a list item applies to your research, read the appropriate section before selecting a response.

Materials & experimental systems

n/a	Involved in the study
☒	☐ Antibodies
☐	☒ Eukaryotic cell lines
☒	☐ Palaeontology and archaeology
☒	☐ Animals and other organisms
☐	☒ Clinical data
☒	☐ Dual use research of concern

Methods

n/a	Involved in the study
☒	☐ ChIP-seq
☒	☐ Flow cytometry
☒	☐ MRI-based neuroimaging

Eukaryotic cell lines

Policy information about cell lines and Sex and Gender in Research

Cell line source(s)	K562 and Kasumi-1 were acquired from the American Type Culture Collection (ATCC).
Authentication	Detection of somatic mutations in TP53 (TP53Q136fs in K562 and TP53R248G in Kasumi-1) and fusion genes (BCR::ABL1 in K562 and RUNX1::RUNX1T1 in Kasumi-1) authenticated the utilized cell lines.
Mycoplasma contamination	The cell lines were not tested for mycoplasma contamination as this was not relevant for the single cell genomic assays in this manuscript.
Commonly misidentified lines (See ICLAC register)	No commonly misidentified cell lines were used in this research.

Clinical data

Policy information about clinical studies

All manuscripts should comply with the ICMJEguidelines for publication of clinical research and a completedCONSORT checklist must be included with all submissions.

Clinical trial registration	NCT02890329
Study protocol	The study protocol can be accessed at clinicaltrials.gov. The study has been described in detail in Garcia et al., Blood 2023 (https://doi.org/10.1182/blood.2022017686). Further information is available from the principle investigator (Jacqueline Garcia at Dana-Farber Cancer Institute Jacqueline_Garcia@DFCI.HARVARD.EDU).<br><br>Relevant information for this research from the clinical study was transplant status (arm A or B) and best objective response (secondary outcome).
Data collection	Anti-leukemic activity was reported as secondary outcome at predetermined timepoints for a maximum of 52 weeks after study entry.
Outcomes	Anti-leukemic activity was assessed as secondary outcome according to 2003 International Working Group criteria for acute myeloid leukemia and 2006 International Working Group criteria for myelodysplastic syndrome
